# Dosimetric evaluation of magnetic resonance imaging based synthetic computed tomography for head and neck photon and proton therapy

**DOI:** 10.1002/acm2.70335

**Published:** 2025-11-27

**Authors:** Marte Kåstad Høiskar, René M. Winter, Natalie Hornik, Mirjam Delange Alsaker, Kajsa Fridstrøm, Sigrun Saur Almberg, Kathrine Røe Redalen

**Affiliations:** ^1^ Department of Physics Norwegian University of Science and Technology Trondheim Norway; ^2^ Section for Biomedical Physics, Department of Radiation Oncology, University Hospital and Medical Faculty Eberhard Karls University Tübingen, Geschwister‐Scholl‐Platz Tübingen Germany; ^3^ Department of Radiotherapy Cancer Clinic St. Olavs Hospital, Trondheim University Hospital Trondheim Norway

**Keywords:** Head and neck cancer, MRI‐based synthetic CT, Proton therapy

## Abstract

**Background:**

Accurate target delineation is essential for precise delivery of proton therapy. A magnetic resonance imaging (MRI)‐only radiotherapy workflow may improve delineations, and thus improve proton therapy, but requires reliable synthetic computed tomography (sCT) for accurate dose calculations.

**Purpose:**

The aim was to evaluate the dosimetric accuracy of commercial software‐based sCT for proton dose calculation for head and neck cancer (HNC), and benchmark it against the accuracy of photon dose calculation on the same sCT.

**Methods:**

MRI and planning CT (pCT) were acquired for 20 HNC patients before receiving photon therapy. A commercial software created MRI‐based sCTs, which were co‐registered to pCTs. For each patient, photon and proton plans based on pCT, prescribing 68 Gy to the target, were created in RayStation and recalculated on sCT. Dose‐volume histogram (DVH) metrics and gamma index (2%/2 mm criteria) were analyzed, comparing pCT‐ and sCT‐calculated dose. Local and global gamma index were calculated for low and high‐dose thresholds. A gamma pass rate (GPR) was calculated for each plan and gamma index type.

**Results:**

For photon and proton plans, the median difference in DVH metrics between pCT‐ and sCT‐calculated dose was < 0.4 Gy with an interquartile range < ± 0.7 Gy for all structures and metrics, except for mean dose to oesophagus (median = −0.3 Gy, range: −2.0 Gy to 0.7 Gy), oral cavity (median = 1.8 Gy, range: 0.6 Gy to 3.0 Gy), and larynx (median = −0.1 Gy, range: −1.5 Gy to 0.6 Gy) for proton plans. The median GPR for photon plans was > 97.7%, while for proton plans it was > 93.3% except for body GPR with low‐dose threshold.

**Conclusion:**

While sCT seems feasible for photon therapy, poorer agreement between pCT‐ and sCT‐calculated proton dose was found. Patient positioning differences between CT and MRI may partly explain the discrepancies between pCT‐ and sCT‐calculated proton dose.

## 1 INTRODUCTION

Each year, new proton centres are being built, making proton therapy more available to cancer patients worldwide. Computed tomography (CT) is currently the primary imaging modality for dose calculation in proton therapy planning. However, due to better soft tissue contrast, magnetic resonance imaging (MRI) is used to more accurately delineate clinical target volumes (CTVs) and organs at risk (OARs).^1^ The MR images need to be co‐registered to the CT images for the delineated contours to be transferred to the CT images. The co‐registration process is associated with uncertainties, due to inaccurate registration algorithms and variation in patient setup between scan sessions, which can result in inaccurate planned dose distribution.^2^, ^3^ This is especially a concern for the treatment of head and neck cancer (HNC) patients due to the large number of anatomical structures and level of movement compared to other tumor sites.^4^ An MRI‐only radiotherapy workflow would remove co‐registration errors and hence improve target delineations and potentially clinical outcome.^5^ Accurate delineations are central to the success of proton therapy, with its finite proton range, to avoid missing the tumor and prevent recurrences. Therefore, an MRI‐only workflow may help proton therapy reach its full potential.

One of the technical challenges with clinical implementation of MRI‐only workflow is reliable synthetic CTs (sCTs) for dose calculations.^5^ The electron density, for photon therapy, and the mass density and tissue properties for proton therapy, is required to perform dose calculations. Since this information is retrieved from CT images, sCTs must be created to utilize an MRI‐only workflow. While many methods to create sCTs from MRI have been explored, deep learning algorithms for sCT generation have shown superior efficiency and accuracy for HNC.^6^, ^7^, ^8^ Most sCT studies use in‐house methods for sCT generation and focus on the performance of the algorithms, and there are few studies that investigate the clinical feasibility of commercially available sCT tools.^9^

An MRI‐only workflow may also facilitate MRI‐guided radiotherapy which can help individualize treatment. Functional MRI, such as diffusion‐weighted and dynamic contrast‐enhanced MRI, can quantify tumor heterogeneity associated with treatment response.^10^ For instance, it can be used to determine aggressive sub‐volumes that require dose escalation to improve patient outcome.^11^ Proton therapy, with its unique Bragg peak characteristics, is suitable for dose escalation and the combination may be an effective treatment.^12^, ^13^, ^14^

MRI‐only and MRI‐guided proton therapy has the potential to improve HNC radiotherapy, but reliable sCTs are required. There are studies that show sCTs are feasible for proton therapy for prostate, brain, and liver,^15^, ^16^, ^17^, ^18^, ^19^ but only Thummerer et al. evaluated sCT for HNC,^20^ which is a more complicated tumor site. Thummerer et al. did, however, use in‐house sCT software, not a commercially available one. The aim of this study was to evaluate the accuracy of MRI‐based sCT for proton dose calculations by comparing it to dose calculation based on planning CT (pCT). Since the commercial software is validated for photon therapy, the accuracy of proton dose calculation on sCT was benchmarked against the accuracy of photon dose calculation on sCT.

## 2 METHODS

### 2.1 Patient population

This study included 20 patients with head and neck squamous cell carcinoma who were treated with photon therapy at St. Olavs Hospital in Trondheim, Norway, in the period 2021–2024. The patient characteristics are found in Table 1. The study was approved by the Regional Committee for Medical Research Ethics in Central Norway (approval number 2019/64744) and all patients gave their written informed consent.

**TABLE 1:** Patient characteristics.

Patients		Age (years)		Number of tumors		Proton plans	
Males	Females	Median	Range	Oropharynx (tonsil/base of tongue)	Larynx	Unilateral	Bilateral
15	5	63	51–75	19 (12/7)	1	2	18

### 2.2 Image acquisition and synthetic CT creation

pCT images and T1‐weighted Dixon‐VIBE images, required to generate sCTs, were acquired in treatment position before photon therapy. The median (interquartile range) time between pCT and MR acquisition was 5 days (4–6.5 days). The Dixon‐VIBE images were acquired using a 2‐point Dixon fat‐water separation technique. The pCTs were single‐energy CTs acquired with contrast on a Somatom CT scanner (Siemens Healthineers, Erlangen, Germany), while the MR images were acquired on a 3T PET/MRI scanner (Biograph mMR, Siemens Healthineers, Erlangen, Germany). The patients were immobilized with a thermoplastic head/neck/shoulder fixation mask and head/neck support cushion both during CT and MRI. To enable radiotherapy‐specific patient positioning and fixation on the PET/MR scanner, a novel radiotherapy‐tailored imaging setup was used.^21^ The setup consisted of a flat tabletop, a baseplate for the mask fixation, and a head and neck coil configuration with three 6‐channel mMR body matrix coils placed on dedicated place holders. In addition, the mMR spine coil was used for posterior neck coverage. The scan parameters for the pCT and Dixon images are listed in Table 2.

**TABLE 2:** Scanning settings for planning CT and Dixon images which were used for synthetic CT generation.

Planning CT	
Acquisition matrix	512 × 512
Number of slices	135–174
Reconstruction in‐plane resolution	0.82 × 0.82–1.37 × 1.37 mm^2^
Slice thickness	2 mm
Peak kilo voltage output	120 kV
Tube current	122 mA
T1‐weighted Dixon‐VIBE MRI	
Scan plane	Transversal
Acquisition matrix	416 × 416
Number of slices	144
Reconstructed in‐plane resolution	1.18 × 1.18 mm^2^
Slice thickness	2 mm
Echo time (in‐phase/out‐of‐phase)	2.57 ms/1.34 ms
Repetition time	4.05 ms
Number of averages	2
Bandwidth	1095 Hz/px
PAT mode	CAIPIRINHA
Accel. Factor 3D	2
Flip angle	9.0 deg
Filter	Distortion correction (3D, from vendor)
Scan time	6:38 min

For each patient, a sCT was generated from the set of four Dixon images, that is, the in‐phase, out‐of‐phase, fat‐reconstruction, and water‐reconstruction image set, using an FDA‐approved neural network‐based software, MRI Planner v2.3 (Spectronic Medical AB, Helsingborg, Sweden).^22^ MRI Planner is trained on Dixon images using a transfer function estimation algorithm. It uses a high degree of data augmentation, meaning it works well for a variety of MR scanners and acquisition parameters. Although another FDA‐approved sCT tool for HNC exists,^23^ MRI Planner is the only software that is not integrated into an MRI scanner and was thus chosen for this study. All sCTs were rigidly registered to the corresponding pCT with an automatic software tool, focusing on the body contour and the bones, in RayStation v12.B (RaySearch Laboratories AB, Stockholm, Sweden) and resampled to the same voxel size as the pCT with 3D Slicer (v5.2.2) using b‐spline interpolation. CTVs and OARs were delineated on pCT by a radiation oncologist with help from MR images. Some of the OARs were generated by artificial intelligence tools in RayStation, adjusted when appropriate, and approved by the oncologist. Delineated contours were further copied from pCT to sCT, except the body, soft tissue, bone, and air contours which were generated for pCT and sCT separately. The body was delineated automatically in RayStation. Soft tissue, bone, and air were defined as regions within the body contour that had Hounsfield unit (HU) between −250 and 250 HU, more than 250 HU, and less than −250 HU, respectively, and were delineated in 3D Slicer (v5.2.2) using thresholding technique.

### 2.3 Comparison between synthetic CT and planning CT

Before analyzing the CTs, the pCTs were pre‐processed in two steps. First, the contrast uptake in the pCT was delineated and its mass density was set to 1 g/cm^3^ (water), to mitigate the effect of contrast‐enhancement in the CT comparison and dosimetric evaluation, since the sCT was generated without contrast. Second, the table and fixation equipment in the pCT was delineated and its mass density set to 0.001 g/cm^3^ (air), as the equipment was not present in the sCT image.

To evaluate the HU accuracy of the sCT, difference maps (pCT minus sCT) and mean absolute error (MAE) between the sCT and the pCT were calculated for the intersection of whole body, soft tissue, bone, and air for each patient.^24^ The same structures were used to assess the spatial‐structural agreement between pCT and sCT by calculating the Dice score, Hausdorff distance (HD), and average symmetric surface distance (ASSD)^25^ for each patient.

ASSD of the body contour was further used as an estimate of the quality of the image registration. Additionally, for each patient, a contour containing the voxels that did not overlap between the body contour of the pCT and sCT was created. Two volumes of the contour were estimated, one including only the areas where photon beams passed through and one where only the proton beams passed through, resulting in beam‐visible misregistration volumes *V*_photon_ and *V*_proton_, respectively.

### 2.4 Photon and proton planning

Volumetric modulated arc therapy plans and intensity‐modulated proton therapy plans were automatically created on pCT in RayStation using in‐house developed scripts, prescribing a median dose of 68 Gy to the high‐risk CTV of primary tumors (CTVp_68_) and lymph nodes (CTVn_68_) and 50 Gy to the elective nodal volumes (CTVe), all in 34 fractions. All plans were created according to local clinical guidelines which follows the DAHANCA protocol^26^ and were approved by an experienced medical physicist. The photon plans consisted of one 6 MV arc using the beam model of an Elekta Versa linear accelerator, planning target volume (PTV)‐margins were 4 mm, and the dose was calculated with the collapsed cone algorithm with 2 mm isotropic dose resolution. To achieve robust photon plans, minimum 98% of the PTVs got minimum 95% of the prescribed dose. Proton plans were planned with five fields (0°, 50°, 160°, 200°, and 310°) for bilateral and four fields (0°, 50°, or 310°, 160° or 200° and 180°) for unilateral irradiation using the beam model of a Varian ProBeam360. A robust beam set‐up was used by restricting each proton field to specific head and neck areas. Additionally, robust optimization with an isotropic position uncertainty of 4 mm and a density uncertainty of ± 3.5% was applied for the proton plans. All proton plans were robustly evaluated and fulfilled the criteria that 99% of the CTVp_68_ and CTVn_68_ and 98% of the CTVe got minimum 95% of the prescribed dose in the worst‐case scenario. The dose was calculated with Monte Carlo simulations, with a maximum statistical uncertainty of 0.5%, and with an isotropic dose resolution of 2 mm. Photon and proton plans based on pCTs were recalculated on the sCTs, without re‐optimization, in RayStation. The recalculated dose distributions were resampled with 3D Slicer using b‐spline interpolation to achieve the same resolution as the pCT‐calculated plans.

### 2.5 Dose evaluation

Dose‐volume histogram (DVH) parameters of CTVp_68_ and OARs were compared between pCT‐ and sCT‐calculated plans. For CTVp_68_, the absolute difference in near‐maximum (*D*_2_) and near‐minimum dose (*D*_99.5_), defined as the dose received by 2% and 99.5% of the target volume, respectively, and the mean dose (*D*_mean_) were calculated. Additionally, the difference in *D*_mean_ for spinal cord, brainstem, left and right parotid, oesophagus, oral cavity, and larynx was calculated, in addition to *D*_2_ for spinal cord and brainstem. The differences were calculated as pCT minus sCT.

Local and global gamma analysis comparing the dose distribution of pCT‐ and sCT‐calculated plans were performed with 2%/2 mm gamma criteria and with a lower dose threshold of 10% and 90% of the prescribed dose. The gamma pass rate was calculated for both the whole body and the union of CTVp_68_, CTVn_68_, and CTVe but only where the contours of pCT and sCT overlapped. The DVH and gamma analyses were performed using in‐house python (v3.11) scripts.

### 2.6 Statistical analysis

To compare DVH metrics of sCT‐calculated dose distributions to the corresponding metrics of pCT‐calculated dose distributions, a Wilcoxon signed rank test was performed for both photon and proton plans.

To evaluate if sCTs affected the photon and proton dose distributions to varying degrees, the DVH metric differences, found between pCT‐ and sCT‐calculated dose distributions, for photon plans were compared to the corresponding metrics for proton plans by a Wilcoxon signed rank test.

After image registration, the pCT and sCT aligned well for some but not all patients due to a difference in patient positioning between CT and MRI scans. Therefore, the impact of change in patient positioning to the difference between pCT‐ and sCT‐calculated dose was evaluated. The Pearson correlations between the local gamma pass rate and both the ASSD of the body contour and the beam‐visible misregistration volumes *V*_photon_ and *V*_proton_, together with their *p* values, were calculated for both photon and proton plans. All Wilcoxon signed rank tests and Pearson correlations were performed in StataMP (v.18) where a *p* value < 0.05 was considered statistically significant.

## 3 RESULTS

### 3.1 Synthetic CT and image registration evaluation

**FIGURE 1:**
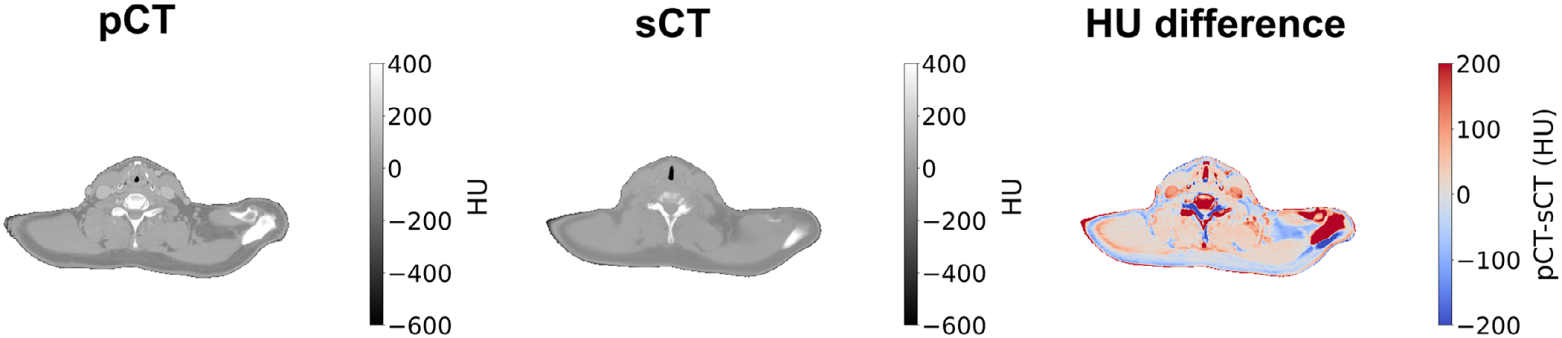
Example of planning CT (pCT), synthetic CT (sCT) and their difference. (a) pCT, (b) sCT, and (c) the difference between them for one patient with well registered pCT and sCT. Here, a slice in the lower head and neck region is shown. HU, Hounsfield unit.

Figure 1 shows an example of pCT, sCT, and the HU difference between them for a slice in the lower head and neck region for one patient laying in similar position during MR and CT acquisition. For the same head and neck slice, the four Dixon images that are used to predict the sCT, shown in Figure 1, are shown in Supplementary Material (Figure S1). Good agreement of the body contour between the sCT and the MR images can be observed.

A similar figure to Figure 1 is found in Supplementary Material (Figure S2), but for a patient with different positioning during MR and CT acquisition. Five out of 20 patients had CTV of lymph nodes outside the body in the sCT due to different patient positioning. Less than 4% of the CTV volumes were outside the body for all but one patient, for which 68% and 54% of the lymph node's GTV and CTV, respectively, was outside the body.

**FIGURE 2:**
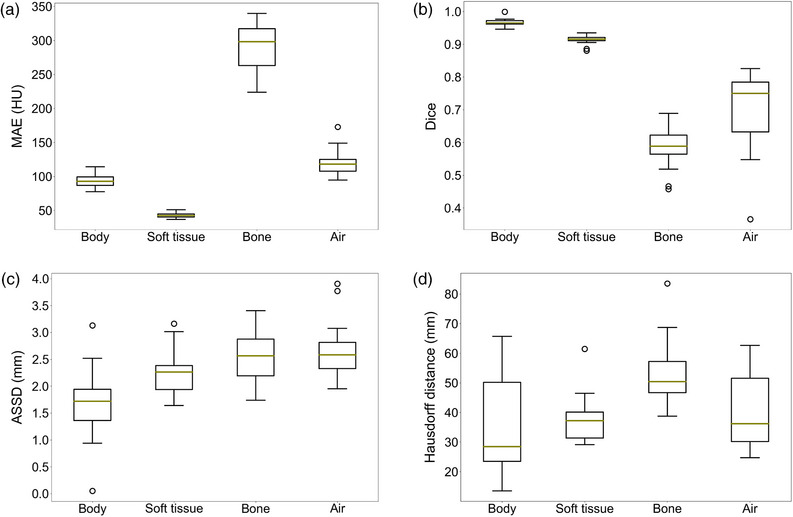
Evaluation of the Hounsfield unit (HU) accuracy of synthetic CT (sCT) and its spatial‐structural agreement with planning CT (pCT). (a) The mean absolute error (MAE) of HU, (b) Dice score, (c) average symmetrical surface distance (ASSD), and (d) Hausdorff distance comparing the sCT to the pCT for the whole body, soft tissue, bone and air. The box extends from the first quartile to the third quartile, while the whiskers extend 1.5 times the interquartile range beyond the box. The green horizontal line is the median, and outliers are shown as circles.

The MAE of HU between the pCT and sCT for the whole body, soft tissue, air, and bone is shown in Figure 2a. The median (interquartile range) MAE over all patients was 93 HU (87–100 HU) for body, 43 HU (40–45 HU) for soft tissue, 298 HU (236–317 HU) for bone, and 118 HU (108–125 HU) for air. The Dice score, ASSD, and HD comparing the body, soft tissue, air, and bone structure in sCT to the equivalent structures in the pCT are presented in Figure 2b–d, respectively. The bone structure, both in terms of HUs and its spatial volume congruence, was the most dissimilar between pCT and sCT (median MAE = 298 HU, median Dice = 0.59). However, its ASSD was similar to other structures (median ASSD = 2.6 mm for bone vs. 2.3  or 2.6 mm for soft tissue and air).

### 3.2 Comparison of DVH metrics

**FIGURE 3:**
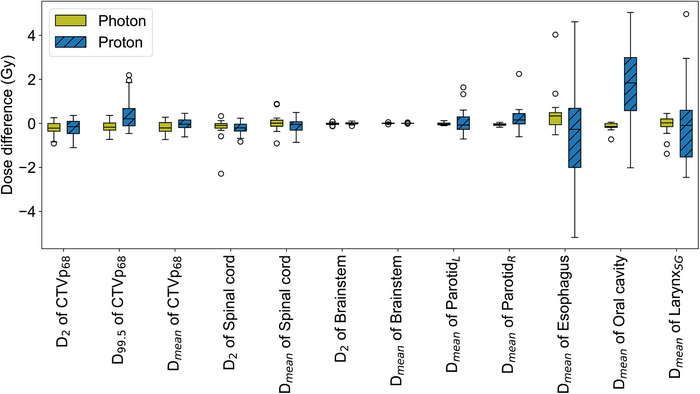
The absolute difference in dose‐volume histogram metrics comparing planning CT‐calculated treatment plans with synthetic CT‐calculated plans for both photons and protons. The high‐risk clinical target volume that is prescribed 68 Gy is called CTVp_68_. *D*_2_ and *D*_99.5_ are the dose given to 2% and 99.5% of the structure's volume, while *D*_mean_ is the mean dose given to the structure. ^*^Statistically significant differences between photon and proton dose differences.

The absolute difference in DVH metrics between pCT‐ and sCT‐calculated plans is shown in Figure 3, for both photons and protons. For photon plans, the difference in *D*_99.5_ of CTVp_68_, *D*_2_ of CTVp_68_ and spinal cord, and *D*_mean_ of right parotid, esophagus, and oral cavity between pCT‐ and sCT‐calculated plans were statistically significant, while for protons only *D*_99.5_ and *D*_2_ of CTVp_68_, *D*_2_ of spinal cord, and *D*_mean_ of oral cavity showed a statistically significant difference (Table S1). However, the dose difference between pCT and sCT were small for most structures and metrics, with a median dose difference within −0.3 Gy to 0.4 Gy and interquartile ranges of < ± 0.7 Gy. This small difference effect of the sCTs on the dose calculation was observed both for photon and proton plans, except for three larger deviations seen for *D*_mean_ of esophagus, oral cavity, and larynx in the case of proton plans.

Between protons and photons, small but statistically significant dose differences (median of proton—photon < −0.4 Gy) were found for *D*_99.5_ of CTVp_68_ (*p* = 0.006) and *D*_mean_ of the right parotid (*p* = 0.022), while a larger dose difference (median of proton minus photon = 1.7 Gy) was found for *D*_mean_ of the oral cavity (*p* < 0.001).

### 3.3 Gamma analysis

**FIGURE 4:**
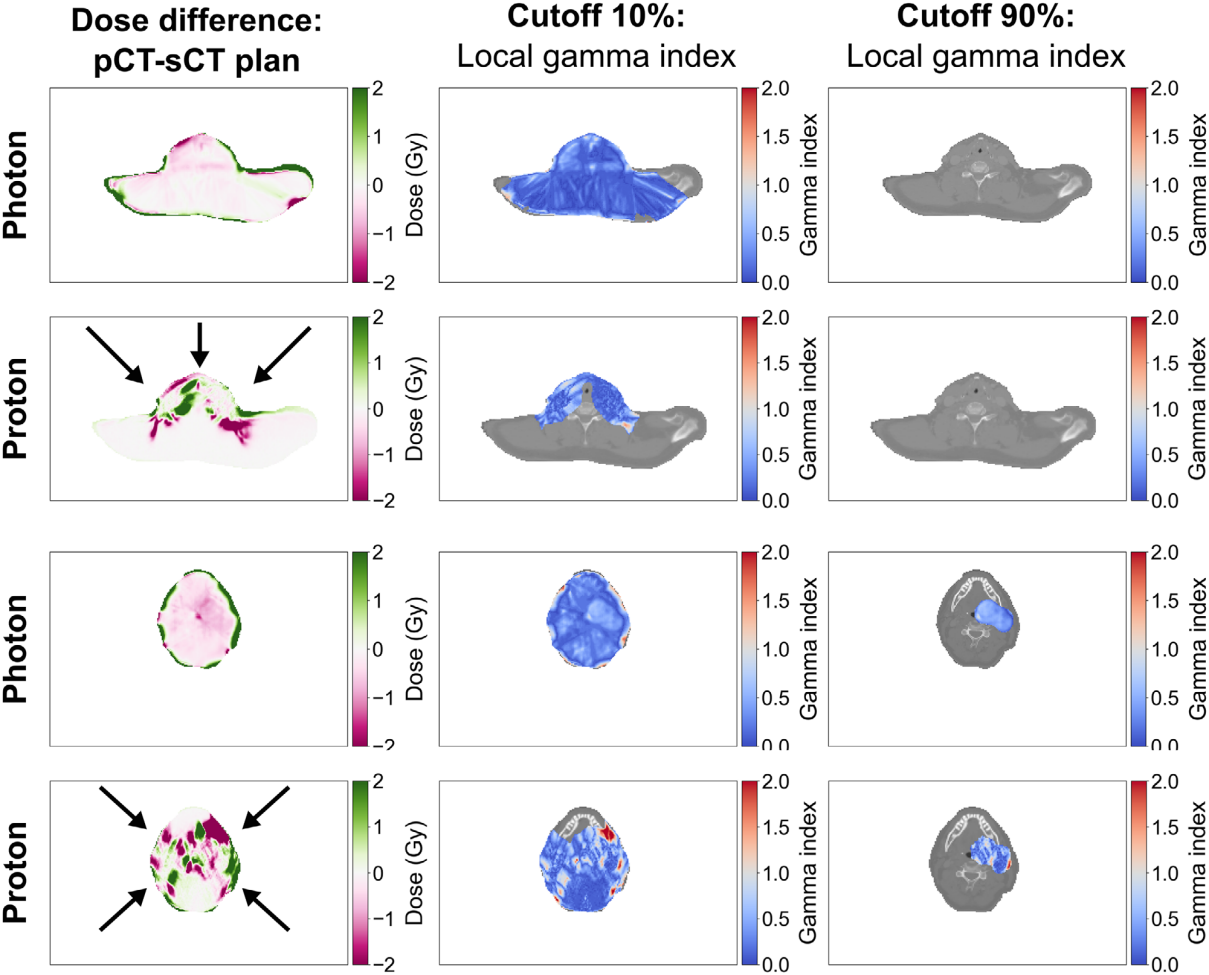
Example of dose difference maps and corresponding gamma index maps for one patient. The dose difference between planning CT (pCT)‐ and synthetic CT (sCT)‐calculated plans (left column) and the local gamma index, comparing pCT‐ and sCT‐calculated plans, calculated with a dose cutoff of 10% (middle column) and 90% (right column) of prescribed dose is shown for both photon (first and third row) and proton (second and fourth row) plans. Here, two different slices of the same patient are given as examples. Black arrows indicate the treatment beam direction in proton plans; for photons, volumetric modulated arc therapy was used.

Figure 4 shows the dose difference between pCT‐ and sCT‐calculated plans for photons and proton plans, together with the corresponding local gamma index for both a dose threshold of 10% and 90%. Red regions in the gamma index plots indicate where the plan fails the gamma criterion of 2%/2 mm. Two different slices, one in the shoulder region and one in the middle of the CTVp_68_, of one patient with similar positioning during CT and MRI acquisition are used as examples in Figure 4. A similar figure can be found in Supplementary Material (Figure S4), though for a patient where the positioning during CT acquisition was poorly reproduced during MRI. For the same patient, examples of dose distributions of pCT‐ and sCT‐based photon and proton plans can be found in Supplementary Material (Figure S3).

**FIGURE 5:**
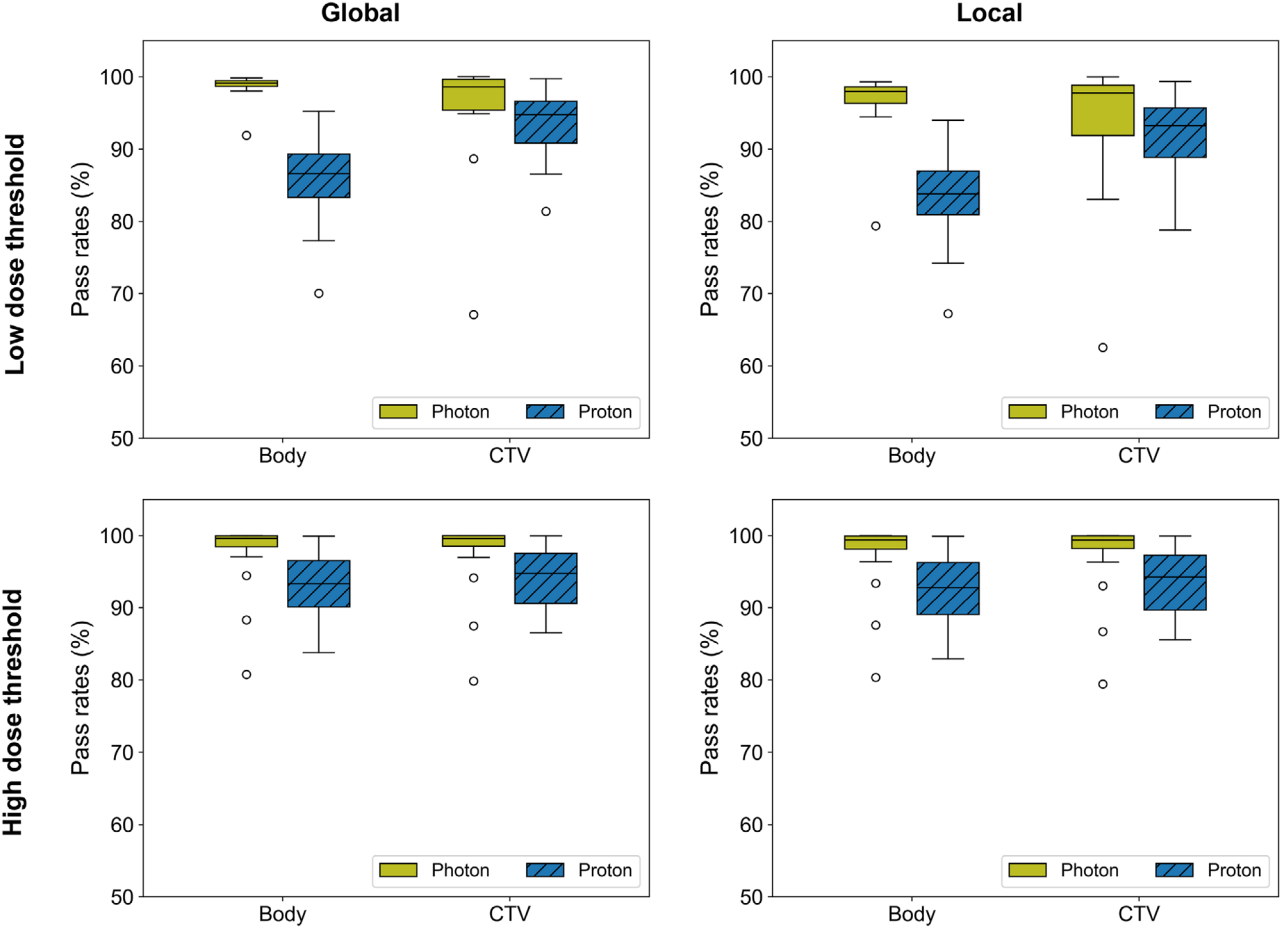
Gamma pass rates for photon and proton plans. Global (left) and local (right) gamma index calculated with gamma criterion of 2%/2 mm and with either low (top row) or high (bottom row) dose threshold. Here, boxplots of the patients’ gamma pass rates are shown for photon and proton plans, for the whole body or the union of clinical target volumes (CTVs).

Figure 5 shows the overall global and local gamma pass rates for the sCT‐ versus the pCT‐calculated plans with a criterion of 2%/2 mm. The results are presented for a low‐ and high‐dose threshold, for both the whole body and the union of CTVs. Overall, the photon plans had a higher gamma pass rate than the proton plans. The pass rate for photons was at least 97.7%/91.9%–98.8% (lowest median/poorest interquartile range as found in the case of local gamma for CTV with low‐dose threshold), whereas the pass rate for protons was at least 93%/89%–96% except for local and global gamma pass rate for body with low‐dose threshold which were below 90% (83.8%/81.0%–86.9% and 87%/83%–89%). The pass rate was similar for the whole body and the CTV union when using a high‐dose threshold. When applying low‐dose threshold, the pass rate was higher for the CTV union than the whole body for protons, while they were similar but more varied for CTV union, for photons.

There was a strong association between the local gamma pass rate and the beam‐visible misregistration volume for both photon and proton plans. The Pearson correlation between the local gamma pass rate for photon plans and *V*_photon_ was −0.60 (*p* = 0.007), while it was −0.73 (*p* < 0.001) between gamma pass rates for proton plans and *V*_proton_, as shown in Supplementary Material (Figure S5). More moderate but significant correlations were also found between the local gamma pass rate and ASSD of the body contour (*r* = −0.64 for photons and −0.49 for protons, Figure S6).

## 4 DISCUSSION

To utilize the full potential of MRI in proton therapy, reliable sCTs for dose calculations are required. This study has shown that it may be feasible to use commercial‐based sCT for proton dose calculation, although the robustness is not as good as it is for photons. The DVH metric analysis showed small dose differences between pCT‐ and sCT‐calculated plans for both photons and protons for all structures, except for the oesophagus, oral cavity, and larynx in the case of protons. The gamma analysis showed a larger difference between pCT‐ and sCT‐calculated plans for proton plans than photon plans, which to some extent may be explained by the strong association between the proton gamma pass rates and the beam‐visible misregistration volumes.

While similar MAE and Dice for body, soft tissue, and air were found by other HNC studies using either rigid^27^ or deformable registration between pCT and sCT,^28^, ^29^, ^30^, ^31^ most studies provided a lower MAE and higher Dice for bone, except the study by Wang et al. that had a higher bone MAE than this study.^32^ Previous studies also found, in agreement with this study, that the largest discrepancies between pCT and sCT, in terms of HUs and Dice, were found in bone. This may be explained by bones producing low signal in MR images, poor registration of shoulders between pCT and sCT and fewer bone voxels available for training of deep learning models, compared to other tissues.

Although the difference in *D*_99.5_ and *D*_2_ of CTVp_68_ between pCT‐ and sCT‐calculated plans was statistically significant for both photons and protons, as well as *D*_mean_ of CTVp_68_ for protons, the interquartile ranges of the dose differences were small and < ± 0.4 Gy and < ± 0.7 Gy for photons and protons, respectively. While the dose differences for photon plans were higher than the dose differences achieved by Palmér et al. using MRI Planner,^9^ they were still clinically acceptable and similar to other sCT studies for photon therapy.^31^, ^33^ There was a statistically significant, though small, difference between photons and protons for D_99.5_ of the CTVp_68_. For four proton plans, the difference in *D*_99.5_ of the CTVp_68_ between pCT‐ and sCT‐calculated plans was > 1.6 Gy. Three of these cases were tumors in the base of the tongue, a region that is more anatomically unstable than other head and neck regions, which may partly explain the discrepancy in *D*_99.5_ of CTVp_68_ for proton plans.

For photon plans, the difference in mean dose between pCT‐ and sCT‐calculated plans was statistically significant for right parotid, oesophagus, and oral cavity, but the interquartile ranges were still < ± 0.5 Gy. Palmér et al. also achieved lower photon dose difference than this study for the OARs.^9^ For proton plans, the interquartile range for the dose difference was < ± 0.5 Gy for all OARs, except for the oesophagus, oral cavity, and larynx. Although the proton dose difference was statistically significant for oral cavity only, oesophagus and larynx also displayed large variation in proton dose difference in Figure 3. Proton plans are often associated with steeper dose gradients compared to photon plans and since the oesophagus, oral cavity, and larynx often lie close behind the CTV, the steep dose gradients at the distal end of the Bragg peak can end up in the OARs. For some patients, difference in patient positioning between CT and MRI acquisition may have caused a displacement in Bragg peak position. Further, all three structures, that is, oesophagus, oral cavity, and larynx, often lie close to regions with air. As described by Figure 2, it varied how well air regions overlapped between pCT and sCT, which may have contributed to a difference in the placement of the Bragg peak between pCT‐ and sCT‐calculated plans. Additionally, differences in patient position may have caused the steep dose gradients, lateral to the beam direction, to shift. Different placement of steep dose gradients may explain why the dose difference is higher and more varied for the oral cavity, oesophagus, and larynx for protons.

A survey performed by Fusella et al. shows that most radiotherapy clinics that use sCTs deem a body gamma pass rate of 95% acceptable for treatment of brain and pelvis, while only a few deem 90% acceptable.^34^ Most clinics used a local gamma criterion of 2%/2 mm and a low‐dose threshold of 10%. For 19 out of 20 patients in this study, the gamma evaluation of photon plans gave clinically acceptable local and global gamma pass rates above 94%, which was similar or better than other studies,^27^, ^28^, ^31^ when calculated with low‐dose threshold and 2%/2 mm criterion. Palmér et al., who also used MRI Planner, achieved a higher gamma pass rate for photons than this study and clinically acceptable pass rates for all patients.^9^ The patient with clinically unacceptable local gamma pass rates, in this study, was an outlier with a local gamma pass rate of 79%. This outlier patient had the most changed positioning between CT and MRI. The gamma pass rates were lower for proton than photon plans. Thummerer et al. are the only ones who have investigated MRI‐based sCT for proton therapy of HNC, and depending on the dose threshold used in their gamma analysis, the gamma pass rates they found were similar or higher than the results in this study.^20^ In contrast to this study, they used deformable image registration to register pCT to sCT, which may be the explanation behind a higher gamma pass rate.

Similar to the DVH analysis, the results of the gamma analyses of patients with tumors placed at the base of the tongue were worse than for those with tonsil tumors. The differentiation between tongue and tonsil tumors was most evident for gamma pass rates with high‐dose threshold, which is more affected by the change in tumor position between CT and MRI scans.

Despite mask fixation, for some patients, the pCT and sCT were poorly registered, due to a change in patient positioning and anatomy between scan sessions, resulting in regions, especially in the shoulder, where the external contour of sCT and pCT did not overlap. When the radiation beam passes through such regions, it results in areas with high‐dose differences and thus fails in the gamma analysis, as visualized in Figure S3 and S4. A stronger association between the gamma pass rate and the beam‐visible misregistration volume was found for protons than photons. Therefore, the misregistration of pCT and sCT may explain the lower gamma pass rates of proton plans. Future studies should ensure more similar patient positioning and less time between scans.

Although different anatomy between pCT and sCT may have led to poorer agreement between pCT‐ and sCT‐calculated proton plans, it should be noted that robust optimization and a robust beam set‐up was used during proton treatment planning. Therefore, some change in patient position was already accounted for to achieve a robust dose coverage of CTVs. This may partly explain why the pass rate for CTV was higher than for the whole body for protons, though the treatment planning goal of homogeneous dose to the CTV is probably the main reason.

Poor registration also led to part of the lymph nodes’ CTV and CTVe being placed outside the body in the sCT for 5 of 20 patients. However, neither of the DVH metric nor gamma analyses were affected because only the primary CTV was evaluated in the DVH metric analysis and only the dose within the intersection of the body contours was considered in the gamma analysis. In this study, rigid image registration was chosen, and an alternative would have been deformable image registration. Although it would remove the discrepancies in the external contour between pCT and sCT and ensure all structures were placed within the body, it would also weaken the integrity of the sCT data as it changes its HUs.

Palmér et al. also used MRI Planner for sCT generation and rigid registration to transfer delineated contours from pCT to sCT before recalculating the photon dose on the sCT.^9^ However, they modified the body contour of the sCT to resemble the body of the pCT, resulting in no discrepancies between the body contour of the pCT and the sCT. This may explain the low‐dose difference in DVH metrics and the high gamma pass rates compared to this study. In an MRI‐only workflow, where there are no image registration errors, the dose distribution of sCT‐calculated plans may therefore be more similar to pCT‐calculated plans than what is observed in this study.

Although discrepancies between the body contours of pCT and sCT were mostly caused by differences in patient positioning, sometimes parts of the shoulder in the MR images were blurry which may have made it difficult for MRI Planner to accurately visualize the shoulder edge in the sCTs. A few other artifacts were also observed. For some patients, there was a larger signal loss in the teeth, most likely due to dental implants. The teeth were still displayed in the sCT, though not always accurately. This may have affected the photon dose, but for protons, the effects should be negligible because the proton beams do not enter through the mouth. For a few patients, zipper artifacts could be observed in the inferior part of the MR images, resulting in some small distortions in the sCT. Since they only occurred in the low‐ or no‐dose region, it had little to no effect on the dose evaluation. However, for one patient, the body contour of the sCT was altered due to zipper artifacts and needed to be modified to achieve a fair comparison between sCT and pCT. In all, one should pay attention to possible artifacts when acquiring MR images for treatment planning and when using MRI Planner.

There were some limitations to this study. The sCT was only evaluated for 20 patients and all but one had oropharynx cancer. Future studies should also include other HNC sites that are commonly treated with proton therapy to evaluate whether sCT is feasible for all HNC patients. Further, the patients were immobilized with fixation masks used for photon therapy during imaging, which differ from masks used for proton therapy. Additionally, pCTs were contrast‐enhanced while sCTs were not. Lalonde et al. and Hwang et al. found that contrast‐enhancement shifted the proton beam range by 3.2 mm for one liver patient^35^ and at median 10 mm for lung patients,^36^ respectively. Therefore, contrast‐enhancement may explain some of the dose discrepancies between pCT‐ and sCT‐calculated dose but only to a small extent since most of the highly contrast‐enhanced areas in the pCTs were removed. Multicenter sCT studies, with more HNC sites and appropriate masks for proton therapy, should be conducted before sCT can be deemed clinically acceptable for proton therapy. Additionally, MRI Planner's deep learning algorithm has trained on single‐energy CTs, whereas dual‐energy CTs can estimate more accurate stopping power ratio.^37^ Therefore, other algorithms that can create synthetic dual‐energy CT from MRI may be a better solution for MRI‐only proton therapy.

In this study, delineated structures were copied from pCT to sCT after rigid image registration to enable recalculation of photon and proton dose on sCT. This made it possible to perform a gamma analysis that evaluated the dose difference between pCT‐ and sCT‐calculated plans on a voxel‐by‐voxel basis. However, by reoptimizing, instead of recalculating on sCT, the image registration error would have been removed and the sCT‐calculated plans would better represent clinical plans in an MRI‐only workflow. A third option would have been to plan on sCT and recalculate on pCT instead of the other way around. While it would not have removed the image registration error, it may have better reflected the true dosimetric impact of using sCT in an MRI‐only workflow. To our knowledge, all studies evaluating the dosimetric accuracy of sCT for proton dose calculation for HNC have, however, planned on pCT and recalculated on sCT. To still be able to compare this study to other studies planning on pCT, it may be beneficial to perform a two‐way validation as done by Masitho et al.,^38^ although the differences between planning on sCT and recalculating on pCT and vice versa were found to be dosimetrically insignificant. In future work, treatment plans will be planned on sCT as well as pCT to investigate whether MRI‐based sCT can be used to compare plans before deciding the best treatment for the patient which could simplify the current radiotherapy workflow.

## 5 CONCLUSION

In this study, we investigated the dosimetric accuracy of MRI‐derived sCT for HNC proton therapy and benchmarked it against photon therapy. While sCT seems feasible for photon therapy, the dosimetric evaluation showed poorer agreement between pCT‐ and sCT‐calculated proton plans. Difference in patient positioning between CT and MRI acquisition may explain some of the discrepancies between pCT‐ and sCT‐calculated plans, especially for protons with its high sensitivity to anatomical changes compared to photons.

## AUTHOR CONTRIBUTIONS

**Marte Kåstad Høiskar**: Conceptualization; formal analysis; investigation; methodology; visualization; writing—original draft; writing—review & editing. **René M. Winter**: Conceptualization; methodology; supervision; writing—original draft; writing—review & editing. **Natalie Hornik**: Methodology; formal analysis; writing—review & editing. **Kajsa Fridstrøm**: Methodology; writing—review & editing. **Sigrun Saur Almberg**: Conceptualization; investigation; formal analysis; methodology; writing—review & editing. **Mirjam Delange Alsaker**: Investigation; formal analysis; writing—review & editing. **Kathrine Røe Redalen**: Conceptualization; funding acquisition; methodology; supervision; writing—original draft; writing—review & editing

## CONFLICT OF INTEREST STATEMENT

The authors declare no conflicts of interest.

## ETHICS STATEMENT

The study was approved by the Regional Committee for Medical Research Ethics in Central Norway (approval number 2019/64744) and all patients gave their written informed consent.

## Supporting information



Supporting Information
