# Supplementary material for: Dosimetric evaluation of magnetic resonance imaging based synthetic computed tomography for head and neck photon and proton therapy
Source: J Appl Clin Med Phys. 2025 Nov 27;26(12):e70335. doi: 10.1002/acm2.70335 (PMC12660051; doi:10.1002/acm2.70335)
Supplement: Supplementary file 1 — Supporting Information [file ACM2-26-e70335-s001.docx]

## Supplementary materials

**MR imaging for synthetic CT generation**

MRI Planner creates synthetic CT (sCT) images from four image types acquired with a Dixon sequence. The Dixon sequence is a fat-water suppression technique and generates four types of images: in-phase, out-of-phase, fat-reconstruction and water-reconstruction images. Suppl. Fig. 1 shows examples of the four image types. The same slice and patient as are used in Fig. 1 is used as an example here.

**
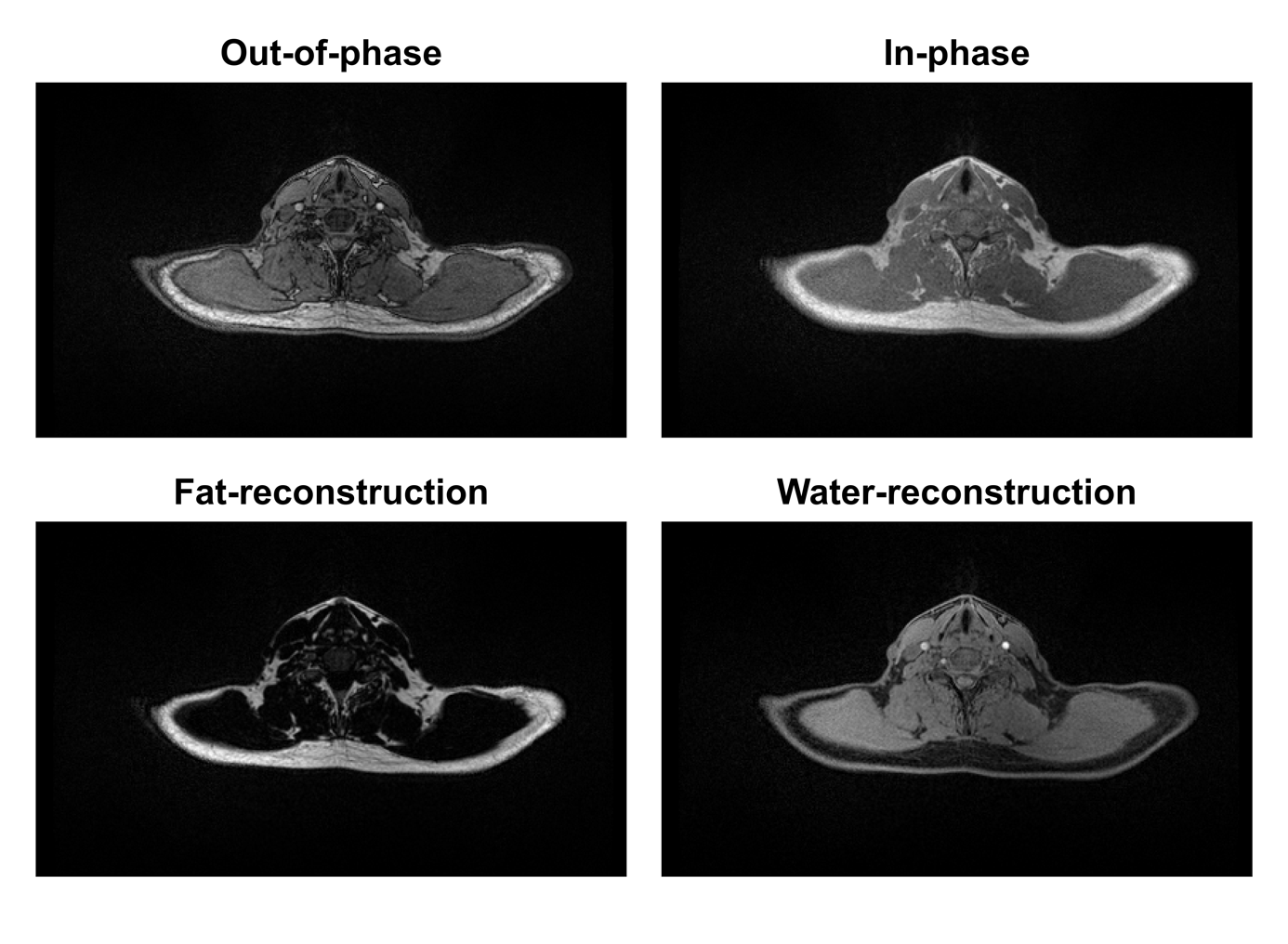
**

Suppl. Fig. 1: Synthetic CTs were generated from the following MR images: out-of-phase (top left), in-phase (top right), fat-reconstruction (bottom left) and water-reconstruction (bottom right), acquired with a Dixon sequence. Here, examples from the same slice of one of the patients are shown.

**Effects of CT registration errors on dose plan evaluation**

The planning CT (pCT) and sCT were rigidly registered to each other. For some patients, it was difficult to achieve a good registration, especially in the shoulder region, due to change in patient positioning. Suppl. Fig. 2 shows an example of a patient with large discrepancy between the external contour of the pCT and sCT. For the same slices of the same patient, Suppl. Fig. 3 shows the dose distribution of pCT- and sCT-calculated photon and proton plans, together with the dose difference (pCT-calculated minus sCT-calculated plan). When radiation beams pass through regions where the external contour largely differs, it results in areas with high dose difference. This seems to affect the proton plans more than the photon plans because photons are delivered with volumetric modulated arc therapy which results in a small dose difference everywhere, rather than smaller regions with high dose difference, as is visualised in Suppl. Fig. 3.

Suppl. Fig. 4 shows an example of how image registration errors can affect the local gamma index. The same dose differences as in Suppl. Fig. 3 is shown in Suppl. Fig. 4, but together with the corresponding local gamma index, with either a dose threshold of 10% or 90%, for both photon and proton plans. Large dose differences, due to discrepancies in external contour, result in regions where the gamma criterion is not met. This also agrees well with the strong association between the local gamma index and the beam-visible misregistration volumes, seen in Suppl. Fig. 5, especially for protons. For the Pearson correlation for photons, one outlier was removed because it dominated the Pearson correlation calculation. There was also a more moderate correlation between the gamma index and the average symmetric surface distance, which is depicted in Suppl. Fig. 6.


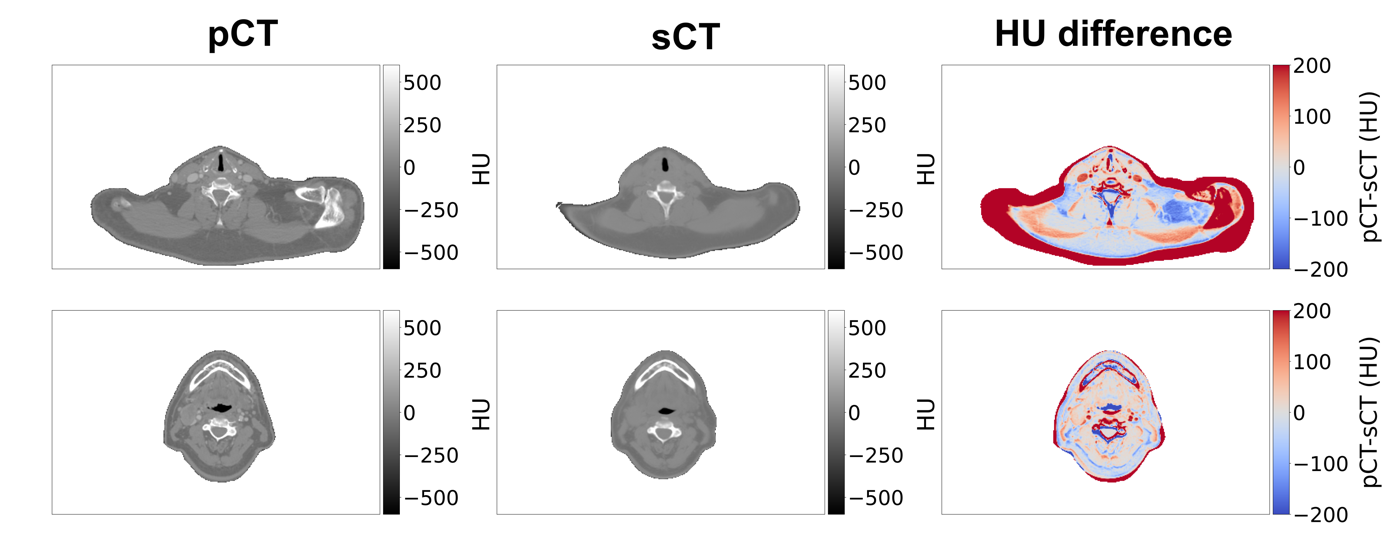


Suppl. Fig. 2: The planning CT (pCT) (left column), synthetic CT (sCT) (middle column) and the difference between them (right column) for two difference slices: one in the shoulder region and the other where a large part of the clinical target volume is, of the same patient with poor image registration of pCT and sCT.


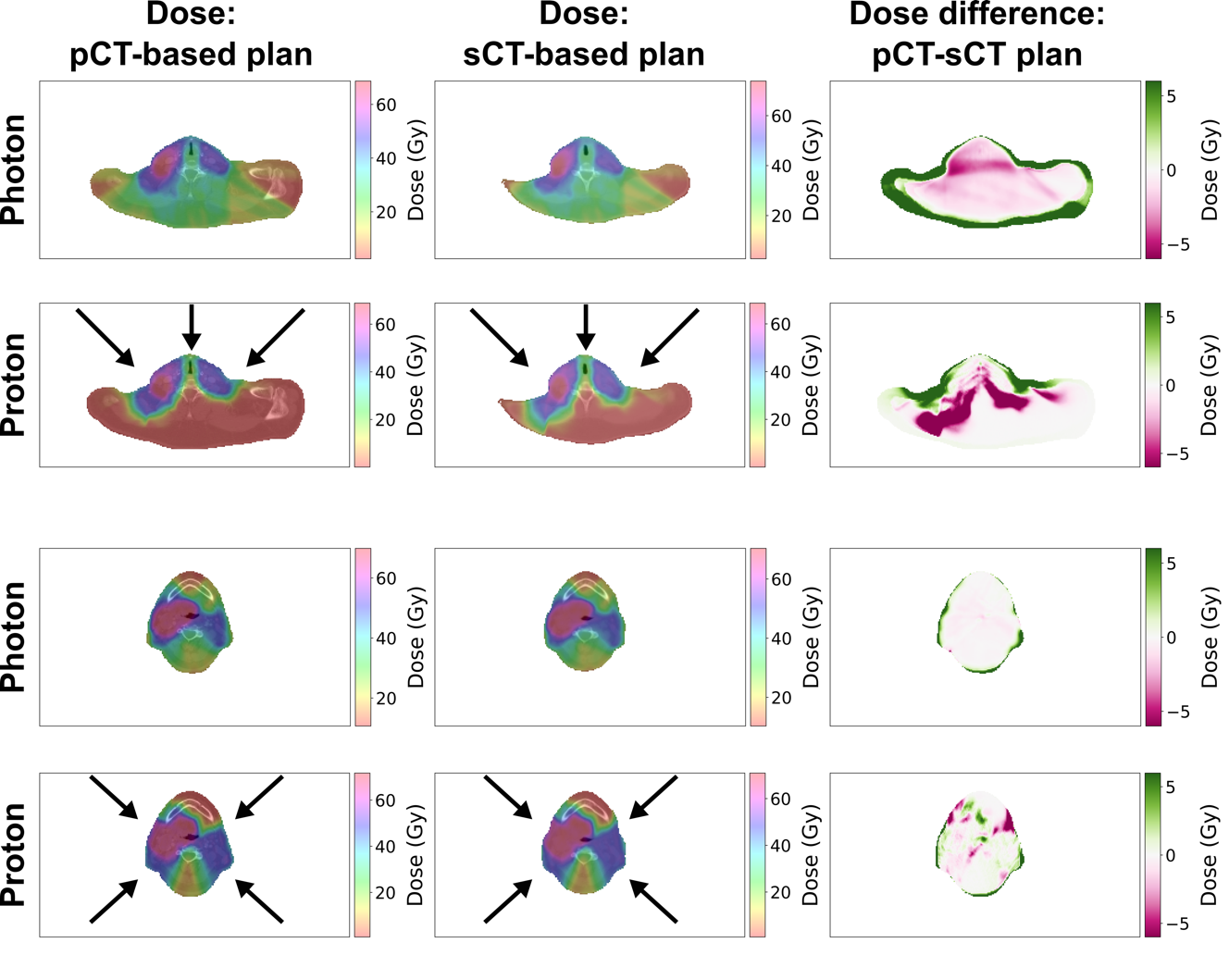


Suppl. Fig. 3: Here is an example of a patient with poorly registered planning CT (pCT) and synthetic CT (sCT), especially in the shoulder region. The pCT-based dose (left column), sCT-based dose (middle column) and the difference between them (right column) for the photon plan (first and third row) and proton plan (second and fourth row) for two different slices: one slice of the shoulder region and one where the clinical target volume lies. The black arrows indicate which directions the proton beam enter the patient.


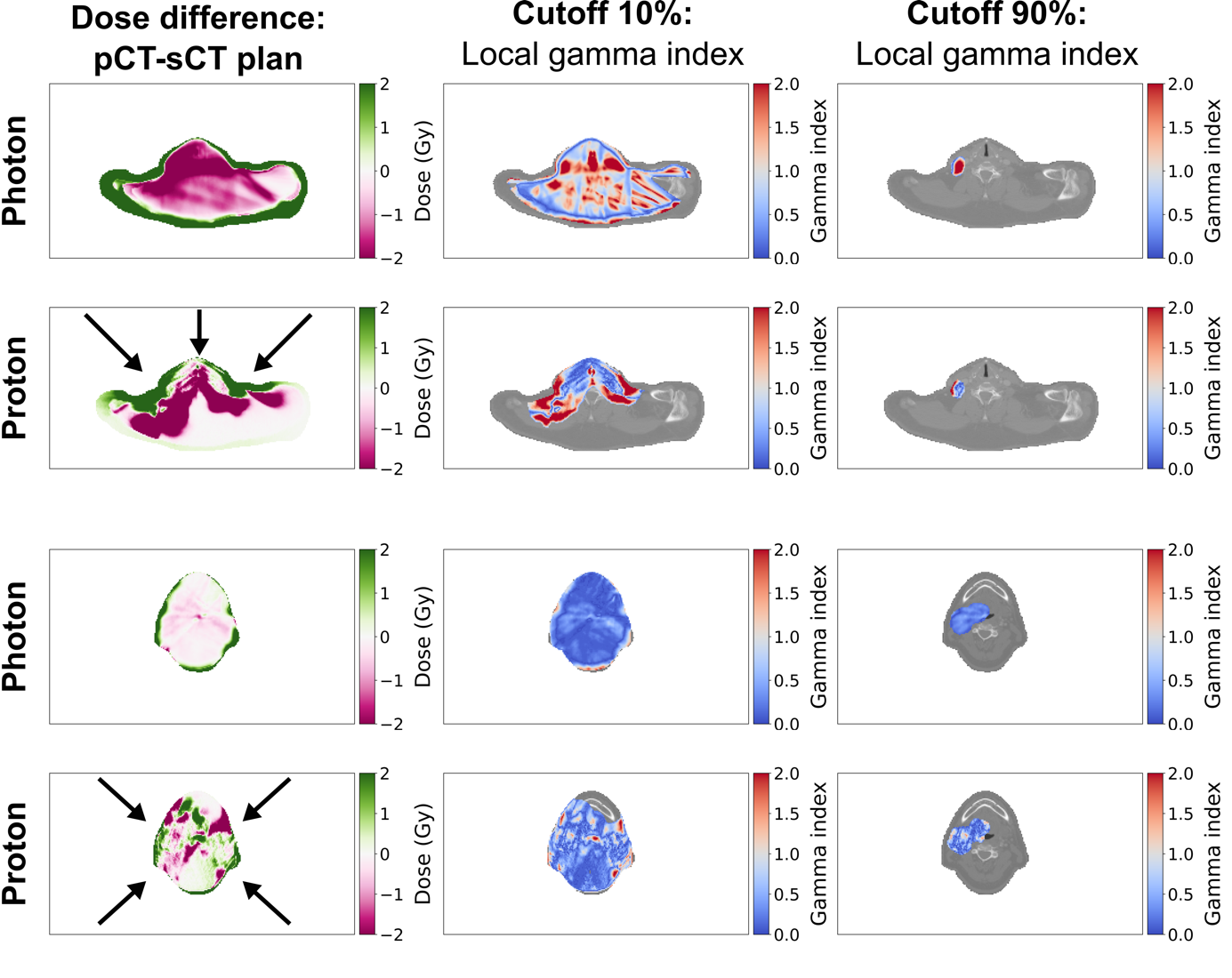


Suppl. Fig. 4: Here, a patient with poorly registered planning CT (pCT) and synthetic CT (sCT) is used as an example. Two different slices of the same patient are depicted: one of the shoulder region and one where the clinical target volume lies. The dose difference (left column) between pCT- and sCT-calculated plans and the local gamma index, with dose threshold of 10% (middle column) and 90% (right column), is shown for both photon (first and third row) and proton (second and fourth row) plans. The gamma index was calculated with a 2%/2mm criterion. The black arrows indicate the directions of the dominant proton beams.

Suppl. Fig. 5: The local gamma pass rate, calculated with low dose threshold and 2%/2mm criterion, of photon (left) and proton (right) plans is plotted against the beam-visible misregistration volumes V_photon_ and V_proton_, respectively. The dashed lines show the linear regression associated with the Pearson correlation of -0.60 (p = 0.007) and -0.73 (p < 0.001) for photons and protons, respectively.

Suppl. Fig. 6: The local gamma pass rate for the photons (left) and proton (right) plans is plotted against the average symmetric surface distance (ASSD) of the body contour. The local gamma index was calculated using a low dose threshold and a 2%/2mm criterion. The dashed lines show the linear regression associated with the Pearson correlation of -0.63 (p = 0.004) and -0.51 (p = 0.02) for photons and protons, respectively.
